# Assessment of vital pulp therapy education in undergraduate dental programs in Saudi Arabia: A cross-sectional study

**DOI:** 10.12669/pjms.41.2.11022

**Published:** 2025-02

**Authors:** Muhammad Qasim Javed, Badi B. Alotaibi, Syed Fareed Mohsin, Ayman Moaz Abulhamael, Usman Anwer Bhatti

**Affiliations:** 1 Muhammad Qasim Javed, FCPS Associate Professor, Department of Conservative Dental Sciences, College of Dentistry, Qassim University, Saudi Arabia; 2 Badi B. Alotaibi Saudi Board (Endodontics), Associate Professor, Department of Conservative Dental Sciences, College of Dentistry, Qassim University, Saudi Arabia; 3 Syed Fareed Mohsin, PhD Department of Oral and Maxillofacial Diagnostic Sciences, College of Dentistry, Qassim University, Saudi Arabia; 4 Ayman Moaz Abulhamael Department of Endodontics, Faculty of Dentistry, King Abdulaziz University, Saudi Arabia; 5 Usman Anwer Bhatti, FCPS Department of Operative Dentistry and Endodontics, Riphah International University Islamabad, Pakistan

**Keywords:** Caries, Dental Education, Endodontics, Permanent Teeth, Vital pulp therapy

## Abstract

**Background & Objective:**

Dental caries remains a paramount public health concern among young children in Saudi Arabia. Vital pulp therapy is a vital component of regenerative endodontics that aims to maintain the vitality of the pulp. This study evaluates the teaching methods used for Vital pulp therapy (VPT) in undergraduate dental programs among Saudi Arabian dental schools.

**Methods:**

This survey was conducted in fifteen dentistry colleges in Saudi Arabia, specifically targeting undergraduate institutions. The survey was administered through a Google form e-questionnaire. The colleges were classified according to their year of establishment. The study evaluated the implementation of VPT in lectures and pre-clinic/simulation sessions and identified the faculty members responsible for conducting these sessions.

**Results:**

All surveyed colleges provided didactic teaching on VPT for permanent teeth. However, only 46.7% of the colleges included practical VPT training in their pre-clinic/simulation curriculum. Among colleges with founding years < 10 years, only 25% offered practical VPT training; however, established institutions over 20 years are more inclined to incorporate VPT into their pre-clinic/simulation sessions. The pre-clinical simulation sessions were mainly conducted by endodontists (85.7%), with a minor involvement from pedodontists (14.3%).

**Conclusion:**

Saudi dental schools should standardize and enhance their VPT curricula to better prepare dental students for contemporary practice. Addressing current deficiencies and adopting the proposed changes will likely improve patient outcomes in vital pulp therapy.

## INTRODUCTION

Vital pulp therapy (VPT) has recently gained popularity as an alternative to root canal therapy (RCT).^1^,^2^ Previously, VPT procedures like pulp capping and pulpotomy were limited to cases of immature permanent teeth judged to have a reversibly inflamed pulp.^3^ While cases with irreversible pulp inflammation were best treated with RCT.^4^ Based on this criteria, any carious pulp exposure was declared unfit for VPT. Consequently, this approach meant the loss of pulp and all its sensory, immune and regenerative benefits in most clinical situations.^5^ However, in 2021, the American Association of Endodontists (AAE) issued a position statement in favour of VPT, broadening the scope of its clinical application to irreversible pulpal inflammations.^6^ According to this statement, a magnified visual assessment of the pulp following haemorrhage control offers a chance to treat cases of varying pulpal inflammation.

This paradigm shift from RCT to VPT is attributed to several factors. Firstly, the improved understanding of pulpal pathology suggests that pulp has areas of varying inflammation that can be objectively viewed under magnification, even when clinical symptoms may indicate irreversible pulpitis.^7^,^8^ Secondly, modern calcium silicates have shown promising clinical results with their favorable properties like alkalinity and inhibition of proinflammatory cytokines.^9^ Lastly, the reported success of VPT and RCT in the literature ranged from 85- 100% and 68-96% respectively.^10^-^15^ Together these factors and the plethora of scientific evidence make VPT a viable treatment option for patients diagnosed with irreversible pulpitis.^6^

While VPT is technically less demanding than RCT, like any other skill dentists need prior training. Unfortunately, most undergraduate training focuses on developing skills for performing RCT. Alarmingly, even in the U.S. most dental schools do not teach VPT on permanent teeth in the simulation clinics.^16^ Given some time has elapsed since the AAE position statement was issued, it will be interesting to see how dental schools in the Kingdom of Saudi Arabia (KSA) teach VPT to their students. Hence, the objective of this study was to evaluate how dental schools in Saudi Arabia educate their students about VPT.

## METHODS

The sample size (SS) was calculated by Raosoft SS calculator.^17^ Fifteen was calculated as adequate SS with total 20 University affiliated dental schools in Saudi Arabia (18 public dental schools and two private dental schools),^18^ 85% confidence level, 10% margin of error and 50% response distribution. The cross-sectional study included 13 public dental schools and two private sector dental schools. The dental schools with at least two graduate batches were included in the study.

### Ethical Approval:

The research was approved by the committee of research ethics at Qassim University (no: 24-84-18).Dated: April 05, 2024.

The questionnaire used was adopted from a previous study conducted at US dental schools.^16^ The Google form e-questionnaire comprising 13 questions in three sections was prepared. The section one comprised of three questions on dental school age, geographic location, and faculty teaching VPT as a technique on permanent teeth during pre-clinic/simulation sessions. The section two consisted of two questions on students’ didactic and practical teaching of VPT on permanent teeth. The section three had eight questions in relation to VPT treatment on permanent teeth. The data collection was initiated by making direct communication (WhatsApp/ phone calls) with relevant faculty members at the target institutes. The faculty members were briefed about the study purpose.

Moreover, they were assured that confidentiality would be maintained and that the names of the participating institutes would not be revealed. Informed consent was obtained from the participating faculty at each institute, and the e-questionnaire link was shared via WhatsApp. Two reminders were sent at weekly intervals. The data collection was carried out from April to May 2024. The results of the study were analyzed by utilizing IBM SPSS, version. 25 (IBM Corp, Armonk, NY) and presented as simple descriptive statistics.

## RESULTS

**Fig.1:**
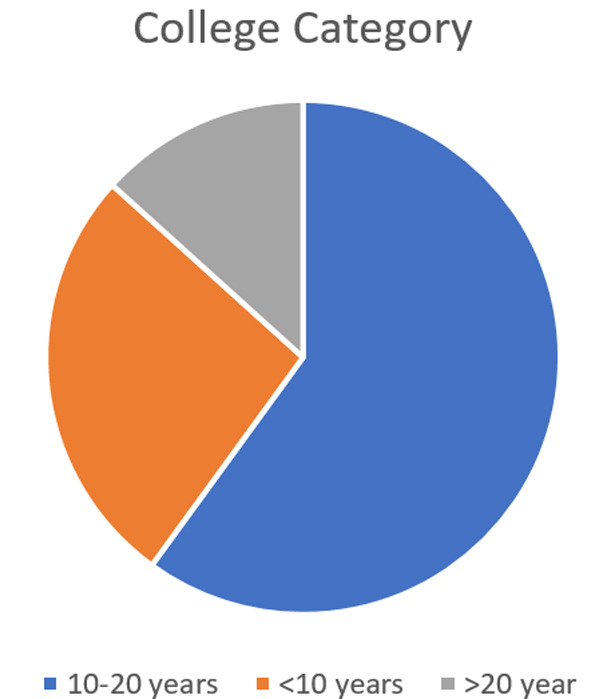
Pie chart depicting number of dental schools in different groups based on the year of establishment.

Fifteen undergraduate (UG) dental colleges in Saudi Arabia were approached to participate in this survey. All the colleges completed the Google form e-questionnaire. The colleges were categorized based on college founding year as “< 10 years” [n=4 (26.7%)], “10-20 years” [n=9 (60%)], and “> 20 years” [n=2 (13.33%)] (Fig.1). All colleges (n=15) noted that students take didactic teaching on permanent teeth VPT. Alternatively, only sesven (46.7%) colleges indicated that they practically teach VPT as a technique on permanent teeth during pre-clinic/simulation sessions. Additionally, one of four UG programs (25%) with founding years < 10 years documented teaching VPT as a technique on permanent teeth during pre-clinic/simulation sessions.

**Fig.2:**
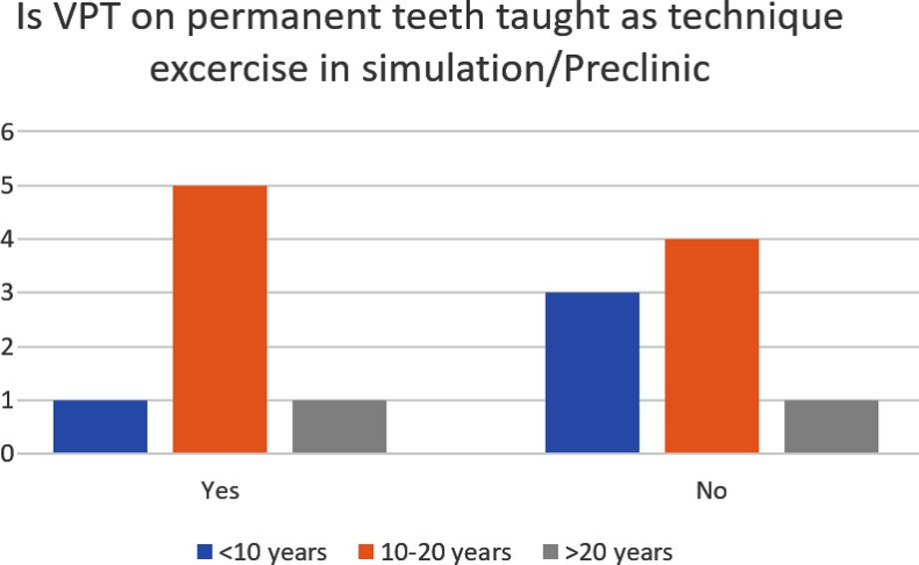
Dental school age versus number of schools within age group teaching vital pulp therapy on permanent teeth as a simulation/pre-clinic technique exercise.

**Fig.3:**
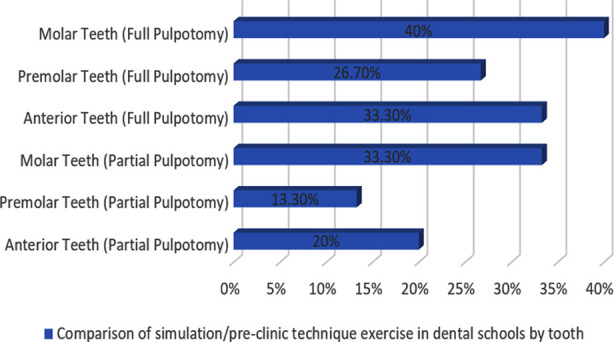
Comparison of simulation/pre-clinic technique exercise in dental schools by tooth.

On the other hand, one out of two (50%) of UG programs with founding years > 20 years and five out of nine (55.6%) of UG programs with founding years of 10-20 years indicated teaching VPT as a technique on permanent teeth during pre-clinic/simulation sessions (Fig.2). A total of 85.7% (n=6) faculty teaching VPT as a technique on permanent teeth during pre-clinic/simulation sessions are Endodontists, with 14.3% (n=1) being Pedodontics. Fig.3, depicts the frequency of various teeth types being utilized to teach the students VPT as a technique on permanent teeth during pre-clinic/simulation sessions in dental colleges.

## DISCUSSION

This study assessed the teaching methods for vital pulp therapy (VPT) in permanent teeth among undergraduate dental programs in Saudi Arabia. The results suggest a notable difference in how and to what degree VPT is included in these programs’ curricula. These variations can significantly impact the readiness of future dental practitioners to offer conservative solutions like VPT to patients with inflamed pulps.

The endorsement of VPT for irreversibly inflamed permanent teeth by renowned endodontic associations indicates an important transition. This decision is the culmination of years of research on the subject.^19^-^21^ While the initial testing of this concept yielded discouraging success rates of no more than 37%, later research showed more promising rates as high as 98%.^22^,^23^ Some authors even reported VPT to be more successful in treating carious pulp exposures than iatrogenic exposures.^24^ Many factors influence the procedure’s success, including inflammation in the pulp, pretreatment pain, mechanical or carious exposure, the size of exposure, tooth age, sealing, and the timing of coronal restorations.^25^ This means that operators need to be knowledgeable and skilled to execute VPT and obtain a favourable outcome.

**Table-I:** Study Questionnaire.^16^

S. No.	Question Statements with answer options
1	How many years has your dental school been in existence?
	Less than 10 years/ 10-20 years/ 20-30 years/ More than 30 years
2	What is the name of your Dental School
3	Which faculty members are involved in teaching Vital Pulp Therapy on permanent teeth as a technique exercise in the simulation/pre-clinic?
	General Dentist/ Endodontist/ Pedodontist/ Prosthodontist/ Other (please specify)
4	Are students given a didactic lecture about Vital Pulp Therapy on permanent teeth?
	Yes/No
5	Is Vital Pulp Therapy on permanent teeth taught as a technique exercise in the simulation/pre-clinic?
	Yes / No
6	In the simulation clinic/pre-clinic, are partial pulpotomies performed on permanent anterior teeth?
	Yes / No
7	In the simulation clinic/pre-clinic, are partial pulpotomies performed on permanent premolar teeth?
	Yes / No
8	In the simulation clinic/pre-clinic, are partial pulpotomies performed on permanent molar teeth?
	Yes / No
9	In the simulation clinic/pre-clinic, are full pulpotomies performed on permanent anterior teeth?
	Yes / No
10	In the simulation clinic/pre-clinic, are full pulpotomies performed on permanent premolar teeth?
	Yes / No
11	In the simulation clinic/pre-clinic, are full pulpotomies performed on permanent molar teeth?
	Yes / No
12	Specify any additional procedures related to Vital Pulp Therapy on permanent teeth performed in the simulation clinic/pre-clinic
13	Are there any other aspects or comments you would like to provide regarding the teaching and practice of Vital Pulp Therapy on permanent teeth in your dental school?

Didactic teaching on VPT for permanent teeth was included in all surveyed dental colleges in Saudi Arabia. This aligns with global standards highlighting the significance of theoretical knowledge in endodontics, as emphasized by the American Association of Endodontists.^6^ Nevertheless, implementing this knowledge in real-world scenarios is still quite restricted, as only 46.7% of colleges have integrated VPT techniques into their pre-clinical or simulation sessions. This gap indicates the necessity for a more practical approach to ensure that students can successfully apply theoretical knowledge to clinical skills, which is essential for their future practice. A study conducted in U.S. dental schools also brought attention to this issue, revealing inconsistencies in the integration of VPT techniques in the curriculum.^16^ The pre-clinical simulation sessions for VPT were mostly instructed by endodontists (85.7%) and a few pedodontists (14.3%). Since VPT is a highly specialized field that demands specific expertise and abilities, it is advantageous to have endodontists in charge of the training. However, including additional pedodontists could offer a more comprehensive viewpoint, especially when managing younger patients.

The study indicates that the founding year of dental colleges influences the extent of practical VPT education. Newer colleges (< 10 years) are less likely to teach VPT in pre-clinical settings than older institutions. This could be attributed to various factors, including resource availability, faculty expertise, and curriculum development. Established colleges with more than 20 years of history have shown a higher likelihood of providing practical VPT training, potentially due to their accumulated experience and resources in dental education. Our findings contrast with another study in which 50% of newer dental schools teach VPT in preclinical/ simulation exercises.^16^

Comparing these findings with a survey of VPT treatment education in U.S. dental schools reveals similar challenges and opportunities for improvement. In the U.S., the incorporation of VPT techniques into the curriculum also varies, with some schools offering extensive practical training while others focus primarily on theoretical knowledge.^16^ This comparison highlights a global need for standardized VPT education emphasizing theory and practice. Establishing a standardized VPT curriculum in all dental schools in Saudi Arabia is necessary to guarantee consistency in teaching approaches and instructional resources. This can be accomplished by collaborating with national dental education bodies and associations. Dental programs should prioritize practical instruction in VPT during pre-clinic and simulation sessions. Participating in practical activities is crucial for students to develop self-assurance and proficiency in executing VPT.

Moreover, using the right materials and techniques for VPT is crucial for its success. The emergence of bioceramic materials such as MTA, and BD has led to enhanced clinical outcomes, which is the primary factor responsible for the effectiveness of VPT for permanent teeth. 7,26 Considering this, dental schools should revise their curricula to incorporate practical teaching to use contemporary bio-ceramic materials in VPT, which have been empirically demonstrated to produce superior clinical results compared to conventional materials like CH. Promoting the ongoing investigation and incorporation of novel methodologies in VPT is advisable. This can be achieved by fostering partnerships with research institutes and implementing ongoing professional development initiatives for academic members. Moreover, it is the need of the hour to create comprehensive national guidelines and policies for VPT education that follow internationally recognized standards. These principles can facilitate the implementation of a standardized method for teaching VPT in all dental schools nationwide.

### Strengths of Study:

The study covered a wide array of undergraduate dentistry programs throughout Saudi Arabia, offering a complete assessment of the present condition of VPT education. The study encompasses colleges with different foundation years and resources, comprehensively representing educational processes. Endodontists’ participation in teaching VPT guarantees that students are instructed by specialists who possess extensive expertise and experience in the subject. This has the potential to improve the standard of education and more effectively equip students for clinical practice. Most importantly, this study provides baseline data on the scope of didactic and clinical training of VPT in Saudi dental schools in light of current AAE recommendations. These findings can guide policymakers to improve the current dental curriculum in Saudi Arabia.

### Limitations:

Although the study involved 15 dental colleges, it is essential to note that this sample may not comprehensively reflect the diverse range of VPT education practices throughout the country. Increasing the sample size could yield a more detailed representation. Certain institutions might overstate their teaching methods, resulting in potential flaws in the research findings.

## CONCLUSION

The results of this study emphasize the necessity for a more uniform and thorough method of teaching VPT in undergraduate dental programs in Saudi Arabia. To enhance the readiness of dental students for the demands of contemporary dentistry practice, dental schools can improve their curriculum by addressing the identified deficiencies and incorporating the suggested modifications. This will ultimately lead to improved patient results in vital pulp therapy.

### Recommendations:

Future studies with a focus on more private and public dental schools of KSA are recommended to give a more comprehensive picture of the current state of didactic/clinical training of VPT at Saudi Dental schools.

### Authors’ Contribution:

**MQJ:** Conceived, designed and did the data collection, data analysis, write up and final approval of the manuscript.

**BBA:** Design, data collection, & reviewed the manuscript.

**SFM, UAB** and **AMA:** Write up, Critical analysis; editing and review.

All authors have approved the final version and are accountable for the integrity of the study.
